# Retrieval Practice Facilitates Judgments of Learning Through Multiple Mechanisms: Simultaneous and Independent Contribution of Retrieval Confidence and Retrieval Fluency

**DOI:** 10.3389/fpsyg.2019.00987

**Published:** 2019-05-03

**Authors:** Xi Chen, Mengting Zhang, Xiaonan L. Liu

**Affiliations:** ^1^Center for Vital Longevity, School of Behavioral and Brain Sciences, University of Texas at Dallas, Dallas, TX, United States; ^2^Institute of Psychology, School of Public Policy, Xiamen University, Xiamen, China; ^3^Department of Psychology, Lehigh University, Bethlehem, PA, United States; ^4^Department of Psychiatry and Behavioral Sciences, University of California, Davis, Davis, CA, United States; ^5^Center for Neuroscience, University of California, Davis, Davis, CA, United States

**Keywords:** judgment of learning, retrieval practice, confidence rating, testing effect, metacognition

## Abstract

Prior studies have shown that predictions of subsequent performance (i.e., Judgments of Learning, JoLs) following tests are more accurate than those following re-study and have suggested that retrieval practice allows people to base their predictions on the current retrieval outcomes so that they assign a higher likelihood of remembering to answers with high confidence. We speculated that other mechanisms, such as retrieval fluency during tests, might also be important for JoLs and that they both offer diagnostic information helping learners to make more accurate JoLs. In the present study, we asked participants to study word-pairs and undergo either a test or re-study trial. Two testing formats (cued-recall and multiple-choice) were administrated for the test condition in two experiments. After the initial test or re-study of the word-pair, participants rated their confidence in the current retrieval accuracy (*test*) or confidence in acquisition (*re-study*), followed by a JoL rating where participants predicted their performance in the final test one day later. The results of both experiments showed that the correlation between JoL ratings and the final accuracy was higher for test trials compared with re-study trials. Moreover, using mediation analyses, we found that this high correspondence was only partially mediated by participants’ confidence in initial tests. Both retrieval reaction time and retrieval confidence simultaneously mediated the correspondence between JoLs and the final accuracy, suggesting that participants were able to correctly base their JoLs on multiple sources of information that are made available through retrieval practice.

## Introduction

Retrieval practice is more beneficial than repeated study: it not only enhances students’ memory performance in a future test (Roediger and Karpicke, 2006; Karpicke and Roediger, 2008) but also helps them make more accurate predictions about future performance (King et al., 1980; Ariel and Dunlosky, 2011). The prospective prediction (i.e., Judgments of Learning, JoLs) reflects the quality of metacognitive monitoring of memory, which is critical for efficient learning (Nelson, 1990; Nelson and Dunlosky, 1991; Benjamin et al., 1998). In an early study (King et al., 1980), participants studied word-pairs and underwent either an initial test or re-study, followed by a JoL rating. Researchers found that participants made more accurate JoLs for the word-pairs that were tested than for the ones re-studied, suggesting proper monitoring associated with retrieval practice.

Some studies have explored why retrieval during a test improves the accuracy of JoLs (Finn and Metcalfe, 2007; Hertzog et al., 2013; Tullis et al., 2013; Serra and Ariel, 2014). One account suggests that participants rely on their memory of test outcomes to make JoLs: participants assign a high likelihood of remembering to the items they believed they answered correctly during the test, and a low likelihood to the ones they did not (Finn and Metcalfe, 2007, 2008). For example, in King’s study (King et al., 1980), participants had higher JoL ratings for successfully recalled items than for those not recalled during tests, suggesting participants’ reliance on retrieval outcome when making JoLs. What is largely unknown, however, is whether confidence in retrieval accuracy is the only factor through which retrieval facilitates JoLs.

Dougherty et al. (2005) attempted to examine whether JoLs following tests simply reflect the confidence in the retrieval outcomes. In their experiment, students made two judgments after the initial test: a retrospective judgment about test accuracy, and a predictive JoL about how confident they were that they would recall the target word in a later test. Results showed that when participants answered correctly in the test, they were highly confident that they had the right answer, but less confident that they would remember it in the future. The authors concluded that the two confidence judgments conveyed different information and suggested that a JoL reflected retrieval confidence plus some “variation,” which could be due to random noise or systematic use of other cues. This result has been replicated in a recent study (Dougherty et al., 2018), together suggesting that multiple sources of information may contribute to JoLs.

Despite the progress in revealing the possibility of other mechanisms underlying JoLs and the benefits of testing, the majority of research has been focusing on isolating contributors, rather than studying JoL as a complex process with multiple bases. It leaves the question open as to what specific information made available through retrieval practice promotes the accuracy of JoLs compared with re-study.

In the present study, we examine whether the high correspondence between JoLs and the final accuracy can be fully attributed to participants’ confidence in their test performance. We speculate that, in addition to confidence in the retrieval outcomes, the speed with which the answer comes to mind might also serve as a clue for making better JoLs (Dunlosky and Metcalfe, 2008). In fact, researchers using general knowledge questions have shown that participants associated the answers that came to mind faster with higher JoLs, even when they were actually harder to remember (Benjamin et al., 1998), suggesting that retrieval fluency may be a potent source for JoLs. Other studies also suggest that JoL increases when the retrieval is easy and fast (Matvey et al., 2001; Koriat and Ma’ayan, 2005; Hertzog et al., 2013). However, whether retrieval fluency truly helps to improve the accuracy of JoL is largely unknown.

This study quantitatively examines the factors that may contribute to more accurate JoLs following tests, adopting a mediational approach. We investigate whether participants’ confidence in retrieval accuracy fully mediates the relationship between the JoLs and the final test accuracy, and examine the hypothesis that both retrieval confidence and retrieval fluency make independent mediational contribution to the relationship between JoLs and the final accuracy. We use two different initial testing formats, cued-recall and multiple-choice, to examine the generalization of results to the situation when the initial test and the final test are in different format, particularly when the initial test is a multiple-choice test, which has been shown to be less effective than a cued-recall test (Glover, 1989; Carpenter and DeLosh, 2006).

## Materials and Methods

### Participants

College students (aged 17–27 years old) participated in this study for course credits. Forty-one participants (mean age = 20.5 years, 32 females) completed the experiment with the cued-recall format. Six of them were excluded due to low accuracy (less than 10%) on the final test. Forty-nine (mean age = 19.7 years, 31 females) completed with the multiple-choice format, ten of which were excluded due to lower than chance level (25%) accuracy. This study was approved by the Ethical Committee, Xiamen University. All participants gave written informed consent in accordance with the Declaration of Helsinki.

### Materials and Procedures

In both experiments, participants studied 120 Chinese word-pairs (Zhang et al., 2018), selected from the Chinese Corpus database ^1^ (see more details in Supplementary Appendix A). Word-pairs were randomly assigned into six lists so that participants only needed to remember 20 word-pairs at a time. During the initial study of each word-pair, participants were instructed to memorize them so that they could recall the target when given the cue. After studying a list, half of the word-pairs were randomly assigned to be *tested*, while the others were *re-studied*. The order of *test* and *re-study* trials was randomly intermixed.

**FIGURE 1:**
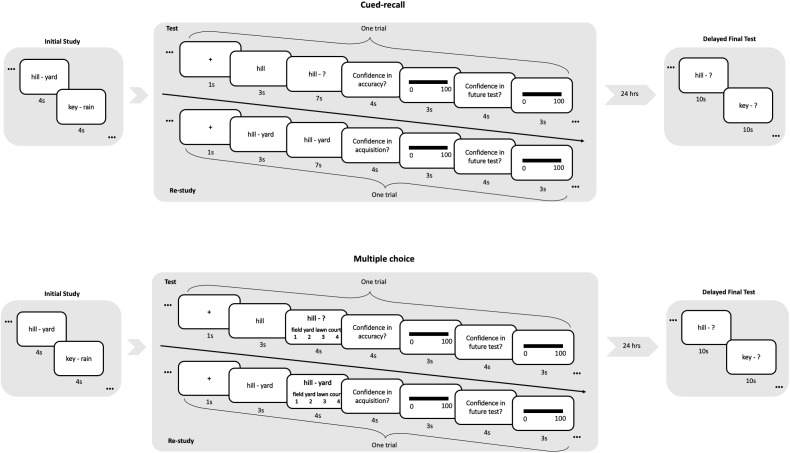
Experimental procedure for the cued-recall and multiple-choice experiments. During both experiments, the cue word was first shown in the center of the screen for 3 s and participants were encouraged to recall the target word. In a cued-recall test, participants were then presented with the cue word on the left and a question mark on the right, and were allowed to enter the answer within 7 s. Because participants were allowed to modify their response during typing, we recorded reaction time as the time participants used to complete their answer. During a multiple-choice practice test, both the cue and four alternatives were presented. The alternatives included the correct target word and three synonyms. Participants had 4 s to enter their choice. No feedback was provided for either condition. A re-study trial presented the intact word-pair, and participants would enter the target word within the same time limit.

We conducted two experiments using two initial testing formats for the *test* condition: cued-recall and multiple-choice. Experimental procedures are detailed in Figure 1. Briefly, for both experiments, the cue word was first shown for 3 s and participants were encouraged to recall the target word. Then, in test trials, participants entered their answer within the next 7 s (in the cued-recall experiment) or enter their choice out of four alternatives in 4 s (in the multiple-choice experiment). Because participants were allowed to modify their response during typing, we recorded reaction time as the time participants used to complete their answer. No feedback was provided for either condition. A *re-study* trial presented the intact word-pair, and participants needed to enter the target word within the same time limit. This ensured that differences between *test* and *re-study* trials would not be simply due to the fact that *test* trials required participants manually entering or selecting the answer.

After each *test* or *re-study* trial, participants rated their confidence in the accuracy of their answer (*test*) or acquisition of the word-pair (*re-study*), on a scale of 0 to 100. Then, participants completed a JoL rating on their confidence, from 0 to 100, that they would be able to remember this word-pair for the final test 24 h later. The final test was administered one day later using a cued-recall test. All cued-recall responses were first corrected for obvious typos before scoring.

## Results

The mean and standard deviation of performance measures are reported in Supplementary Appendix B. Gamma correlations and paired-sample *t*-tests were conducted in SPSS version 25. For the cued-recall experiment, participants’ learning benefited from testing and had higher final accuracy in the *test* condition (*mean* = 0.332, *SD* = 0.146) than in the *re-study* condition (*mean* = 0.295, *SD* = 0.150), *t(34)* = 2.08, *p* = 0.045, as expected. Participants also had a higher gamma correlation between JoLs after *test* trials and final test accuracy *(G* = 0.655, *SD* = 0.128) than the correlation for *re-study* trials (*G* = 0.338, *SD* = 0.233), *t(34)* = 8.773, *p* < 0.001, suggesting that retrieval practice indeed enhanced JoL accuracy. Similar results were also found in the multiple-choice experiment: higher final accuracy (*test*: *mean* = 0.282, *SD* = 0.113; *re-study*: *mean* = 0.223, *SD* = 0.119; *t*(*38)* = 5.299, *p* < 0.001) and higher JoL-final accuracy correlation (*test*: *G* = 0.282, *SD* = 0.113; *re-study*: *G* = 0.223, *SD* = 0.119; *t(38)* = 3.034, *p* = 0.004) for *test* than for *re-study* trials.

**FIGURE 2:**
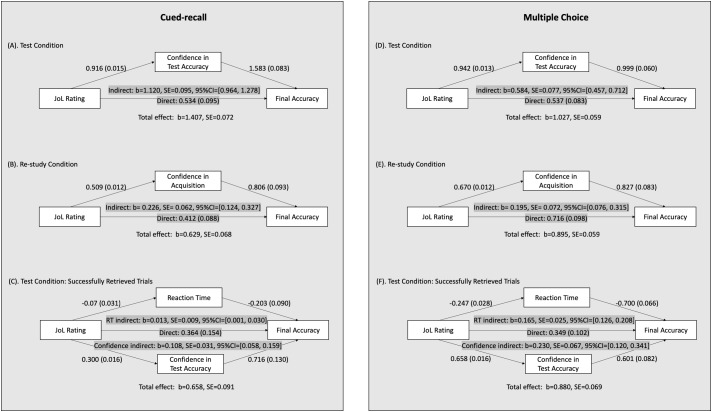
Results from mediational analyses revealed multiple mechanisms underlying the correspondence between JOL ratings and final accuracy. In the cued-recall experiment, **(A)**, for the *test* condition, the mediation of confidence in retrieval accuracy was significant, but did not fully attenuate the relationship between JOL ratings and final accuracy. **(B)** Confidence in acquisition in the *re-study* condition also mediated the relationship between JOL ratings and final accuracy, but the effect was numerically smaller compared with the test condition. **(C)** Both retrieval confidence and reaction time during successful retrievals significantly mediated the relationship simultaneously. **(D–F)** The experiment with multiple-choice testing format showed consistent results.

In the primary mediational analyses, we first examined whether participants relied on the confidence in their retrieval accuracy when making JoLs. Mediation analyses were performed based on mixed model logistic regression using lme4 and RMediation packages in the R Statistical Environment (MacKinnon et al., 2007; Tofighi and MacKinnon, 2011; Bates et al., 2014; R Core Team, 2016). We first modeled the relationship between JoL ratings and the final test performance while including the subject-specific intercepts as the random effect, and then examined the mediation effect of retrieval confidence in this relationship. The analysis revealed that, for the cued-recall experiment, participants’ confidence in the test accuracy significantly mediated JoL’s correspondence with the final performance (Figure 2A), confirming that participants made predictions about their future performance based on their confidence in the practice test. In addition, the mediation effect in the *test* condition was numerically larger than the mediation of acquisition in the *re-study* condition: for *test* trials, indirect/total effect was 79.6%, whereas the ratio was 35.9% for *re-study* trials (Figure 2B), increasing the likelihood that participants made better JoLs for *test* trials than for re-study trials because participants were able to make JoLs based on their confidence in retrieval performance in the *test* condition. Similar patterns of results were also found in the multiple-choice experiment (Figure 2D,E).

Importantly, including the mediator of confidence did not fully mediate the relationship between JoL ratings and the final accuracy, indicated by a significant direct effect (Figure 2A), suggesting that other factors also account for a large portion of the variance in the relationship. We suspected that reaction time also contributed to the relationship and indeed found that reaction time of successfully retrieved trials^2^ was also a significant mediator (Supplementary Appendix C).

In the final set of analyses, we modeled both mediators simultaneously to estimate their independent mediation effects. This was examined due to the fact that trials with longer reaction time were also related to lower confidence rating in retrieval accuracy (*G* = –0.193, *SD* = 0.212, one-sample *t*(34) = −5.375, *p* < 0.001). It is possible that the two mediators shared substantial variance and thus both showed significant effects. The final model showed that both variables significantly mediated the relationship between JoL ratings and the final accuracy (Figure 2C), suggesting simultaneous mediational roles of both retrieval confidence and retrieval fluency. We noted that the effect of reaction time was significant but small in magnitude (indirect/total effect = 2.0%), probably because reaction time of a cued-recall response is generally complex and may be affected by factors in addition to retrieval fluency (e.g., the length of the target word, self-correction during entering, etc.), possibly suggesting that reaction time in cued-recall tests may be a useful but less sensitive measure of retrieval fluency.

Finally, in the multiple-choice experiment, we observed that retrieval accuracy and reaction time were significantly negatively correlated (*G* = –0.370, *SD* = 0.141, one sample *t*(38) = –16.319, *p* < 0.001) and that they both simultaneously and independently mediated the relationship between JoLs and the final accuracy (Figure 2F). These results suggest the generalization of findings for two different initial testing formats, and confirm that retrieval confidence and retrieval fluency both serve as clues to help participants make more accurate JoLs, regardless of the testing format. Meanwhile, we noted that including both mediators still did not fully attenuate the relationship between JoLs and the final accuracy, suggesting that other mechanisms may also contribute to the relationship.

## Discussion

The goal of the present study was to examine the benefits of retrieval practice for JoLs and the factors that may contribute to more accurate JoLs following a test. Consistent with previous findings (Finn and Metcalfe, 2007, 2008), we showed that participants relied on the confidence in their retrieval outcomes when making JoLs. Using a mediational approach, we provided direct evidence that this heuristic indeed helped people to give more accurate JoLs. Moreover, we showed that participants’ confidence in their retrieval performance could not fully explain how they were able to achieve high correspondence between JoLs and their final performance, suggesting that multiple factors provided useful information that helped in making better predictions.

A novel contribution of the study is that it complements the understanding of JoLs by showing strong evidence of multiple mechanisms underlying test-related benefits of JoLs. Recent studies have discussed the possibility that multiple cues might contribute to the processes of making accurate JoLs (Dougherty et al., 2005; Hertzog et al., 2013; Serra and Ariel, 2014). The present study demonstrates that people associate fluent retrievals with higher JoL and that this strategy helps to make more accurate JoLs. In addition, the reliance on both retrieval confidence and fluency was observed for both recognition and cued-recall tests. This consistent pattern of results suggests that participants actively monitor their retrieval processing and use diagnostic information made available through retrievals when evaluating and predicting the learning progress.

In fact, researchers have proposed a two-process account (Liu and Reder, 2016; Liu et al., 2018) for the testing effect, emphasizing that individuals undergo a re-encoding process through re-exposure to the correct answer after successful retrievals (post-retrieval re-encoding), in addition to the retrieval attempt process. This post-retrieval re-encoding process also involves metacognitive monitoring and self-evaluation (Johansson and Mecklinger, 2003; Bai et al., 2015; Zhang et al., 2018) and can further enhance testing-related benefits (Liu and Reder, 2016). In addition, a prior fMRI study showed that testing, compared with re-study, involved more monitoring and working memory-related brain activity (Liu et al., 2014). Altogether, the reliance on retrieval outcomes when making JoLs might reflect the metacognitive evaluation of the quality of the retrieval attempt process. The reliance on retrieval fluency might reflect the monitoring of the amount of working memory resources available during the post-retrieval re-encoding process. Proper monitoring may improve the accuracy of performance prediction by accurately assessing the potential effectiveness of the post-retrieval re-encoding process.

Finally, these findings have important educational implications. Our findings suggest that students may benefit from practice tests prior to an exam, which not only improve their exam performance, but also allow for better metacognitive monitoring based on their subjective experience during the practice test.

## Ethics Statement

This study was approved by the Ethical Committee, Xiamen University. All participants gave written informed consent in accordance with the Declaration of Helsinki.

## Author Contributions

XL developed the study concept and design. XC and XL drafted the manuscript. All authors contributed to data acquisition, analysis, and interpretation of data, provided critical revisions of the manuscript for important intellectual content, and approved the final version of the manuscript.

## Conflict of Interest Statement

The authors declare that the research was conducted in the absence of any commercial or financial relationships that could be construed as a potential conflict of interest.

## Footnotes

1 www.cncorpus.org

2 Incorrect trials were excluded because reaction time during incorrect responses may be confounded by many factors that do not reflect memory processes.

## References

[B1] Ariel R. Dunlosky J. (2011). The sensitivity of judgment-of-learning resolution to past test performance, new learning, and forgetting. *Mem. Cogn.* 39 171–184. 10.3758/s13421-010-0002-y 21264621

[B2] Bai C.-H. Bridger E. K. Zimmer H. D. Mecklinger A. (2015). The beneficial effect of testing: an event-related potential study. *Front. Behav. Neurosci.* 9:248. 10.3389/fnbeh.2015.00248 26441577 PMC4584999

[B3] Bates D. Maechler M. Bolker B. Walker S. (2014). lme4: linear mixed-effects models using Eigen and S4. *R Packag. Version* 1 1–23.

[B4] Benjamin A. S. Bjork R. A. Schwartz B. L. (1998). The mismeasure of memory: when retrieval fluency is misleading as a metamnemonic index. *J. Exp. Psychol. Gen.* 127:55. 10.1037//0096-3445.127.1.55 9503651

[B5] Carpenter S. K. DeLosh E. L. (2006). Impoverished cue support enhances subsequent retention: support for the elaborative retrieval explanation of the testing effect. *Mem. Cogn.* 34 268–276. 16752591 10.3758/bf03193405

[B6] Dougherty M. R. Robey A. M. Buttaccio D. (2018). Do metacognitive judgments alter memory performance beyond the benefits of retrieval practice? A comment on and replication attempt of Dougherty, Scheck, Nelson, and Narens (2005). *Mem. Cogn.* 46 558–565. 10.3758/s13421-018-0791-y 29368228

[B7] Dougherty M. R. Scheck P. Nelson T. O. Narens L. (2005). Using the past to predict the future. *Mem. Cogn.* 33 1096–1115. 10.3758/BF03193216 16496729

[B8] Dunlosky J. Metcalfe J. (2008). *Metacognition.* Thousand Oaks, CA: Sage Publications.

[B9] Finn B. Metcalfe J. (2007). The role of memory for past test in the underconfidence with practice effect. *J. Exp. Psychol. Learn. Mem. Cogn.* 33:238. 10.1037/0278-7393.33.1.238 17201565

[B10] Finn B. Metcalfe J. (2008). Judgments of learning are influenced by memory for past test. *J. Mem. Lang.* 58 19–34. 10.1016/j.jml.2007.03.006 19079570 PMC2836879

[B11] Glover J. A. (1989). The “testing” phenomenon: not gone but nearly forgotten. *J. Educ. Psychol.* 81:392.

[B12] Hertzog C. Hines J. C. Touron D. R. (2013). Judgments of learning are influenced by multiple cues in addition to memory for past test accuracy. *Arch. Sci. Psychol.* 1:23. 10.1037/arc0000003 25914865 PMC4405771

[B13] Johansson M. Mecklinger A. (2003). The late posterior negativity in ERP studies of episodic memory: action monitoring and retrieval of attribute conjunctions. *Biol. Psychol.* 64 91–117. 10.1016/S0301-0511(03)00104-2 14602357

[B14] Karpicke J. D. Roediger H. L. (2008). The critical importance of retrieval for learning. *Science* 319 966–968. 10.1126/science.1152408 18276894

[B15] King J. F. Zechmeister E. B. Shaughnessy J. J. (1980). Judgments of Knowing - the Influence of Retrieval Practice. *Am. J. Psychol.* 93 329–343.

[B16] Koriat A. Ma’ayan H. (2005). The effects of encoding fluency and retrieval fluency on judgments of learning. *J. Mem. Lang.* 52 478–492.

[B17] Liu X. L. Liang P. Li K. Reder L. M. (2014). Uncovering the neural mechanisms underlying learning from tests. *PLoS One* 9:e92025. 10.1371/journal.pone.0092025 24647122 PMC3960161

[B18] Liu X. L. Reder L. M. (2016). fMRI exploration of pedagogical benefits of repeated testing: when more is not always better. *Brain Behav.* 6:e00476. 10.1002/brb3.476 27458542 PMC4875931

[B19] Liu X. L. Tan D. H. Reder L. M. (2018). The two processes underlying the testing effect–Evidence from Event-Related Potentials (ERPs). *Neuropsychologia* 112 77–85. 10.1016/j.neuropsychologia.2018.02.022 29474894

[B20] MacKinnon D. P. Fairchild A. J. Fritz M. S. (2007). Mediation analysis. *Annu. Rev. Psychol.* 58 593–614. 10.1146/annurev.psych.58.110405.085542 16968208 PMC2819368

[B21] Matvey G. Dunlosky J. Guttentag R. (2001). Fluency of retrieval at study affects judgments of learning (JOLs): an analytic or nonanalytic basis for JOLs? *Mem. Cognit.* 29 222–233. 11352205 10.3758/bf03194916

[B22] Nelson T. O. (1990). Metamemory: a theoretical framework and new findings. *Psychol. Learn. Motiv.* 26 125–173.

[B23] Nelson T. O. Dunlosky J. (1991). When people’s judgments of learning (JOLs) are extremely accurate at predicting subsequent recall: the “delayed-JOL effect.” *Psychol. Sci.* 2 267–271. 10.1037//0096-3445.127.1.55

[B24] R Core Team (2016). *R: A Language and Environment for Statistical Computing.* Vienna: R Core Team.

[B25] Roediger H. L. Karpicke J. D. (2006). Test-enhanced learning: taking memory tests improves long-term retention. *Psychol. Sci.* 17 249–255. 10.1111/j.1467-9280.2006.01693.x 16507066

[B26] Serra M. J. Ariel R. (2014). People use the memory for past-test heuristic as an explicit cue for judgments of learning. *Mem. Cogn.* 42 1260–1272. 10.3758/s13421-014-0431-0 24898119

[B27] Tofighi D. MacKinnon D. P. (2011). RMediation: an R package for mediation analysis confidence intervals. *Behav. Res. Methods* 43 692–700. 10.3758/s13428-011-0076-x 21487904 PMC3233842

[B28] Tullis J. G. Finley J. R. Benjamin A. S. (2013). Metacognition of the testing effect: guiding learners to predict the benefits of retrieval. *Mem. Cogn.* 41 429–442. 10.3758/s13421-012-0274-5 23242770 PMC3602315

[B29] Zhang M. Chen X. Liu X. L. (2018). Confidence in accuracy moderates the benefits of retrieval practice. *Memory* 27 548–554. 10.1080/09658211.2018.1529796 30295147

