# Supplementary material for: Retrieval Practice Facilitates Judgments of Learning Through Multiple Mechanisms: Simultaneous and Independent Contribution of Retrieval Confidence and Retrieval Fluency
Source: Front Psychol. 2019 May 3;10:987. doi: 10.3389/fpsyg.2019.00987 (PMC6509741; doi:10.3389/fpsyg.2019.00987)

Supplementary Material

Appendix A. Study materials

A pool of 600 Chinese words was first selected from the Chinese Corpus (www.cncorpus.org) database with the following criteria: (1) the word consists of two Chinese characters, and (2) the word frequency (appearance among every 20 million characters) is in the range of 0.0025% to 0.0103% (medium to high frequency). Next, a total of 240 words were randomly selected from the pool, and randomly arranged into 120 word-pairs. The word-pairs were then randomly assigned to either the test or the restudy condition and divided into six lists, so that participants only needed to remember 20 word-pairs at a time.

Appendix B. Mean and standard deviation of performance measures

Condition	Performance			Confidence Rating		Gamma Correlation	
	Initial Test Accuracy	Initial Test RT for Correct Trials (ms)	Final Accuracy	Accuracy (*Test*) or Acquisition (*Restudy*)	JoL	Retrieval Confidence - Initial Test Accuracy	JoL - Final Accuracy
				Cued-recall Experiment			
Test	.546 (.165)	3056.62 (661.31)	.332 (.146)	.540 (.136)	.359 (.133)	.911 (.101)	.655 (.128)
Re-study	-	3209.31 (645.43)	.295 (.150)	.662 (.153)	.421 (.148)	-	.338 (.233)
				Multiple-choice Experiment			
Test	.765 (.127)	1697.07 (217.74)	.282 (.113)	.653 (.120)	.449 (.162)	.783 (.133)	.282 (.113)
Re-study	-	1423.15 (210.25)	.223 (.119)	.713 (.141)	.493 (.181)	-	.223 (.119)

Appendix C. Reaction time for successfully retrieved trials mediated the relationship in the *test* condition between JOL rating and final accuracy in both the cued-recall experiment and the multiple-choice experiment.

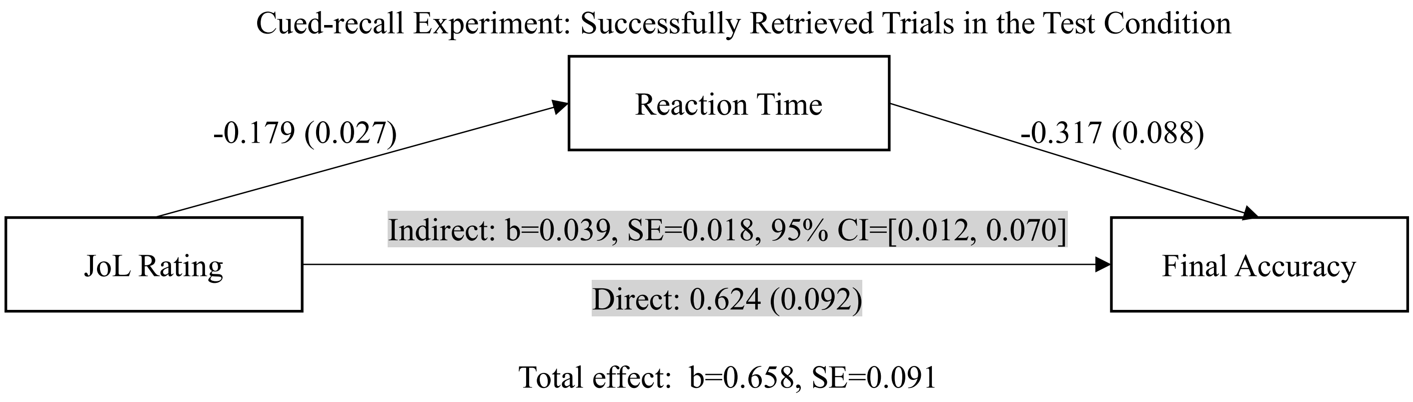

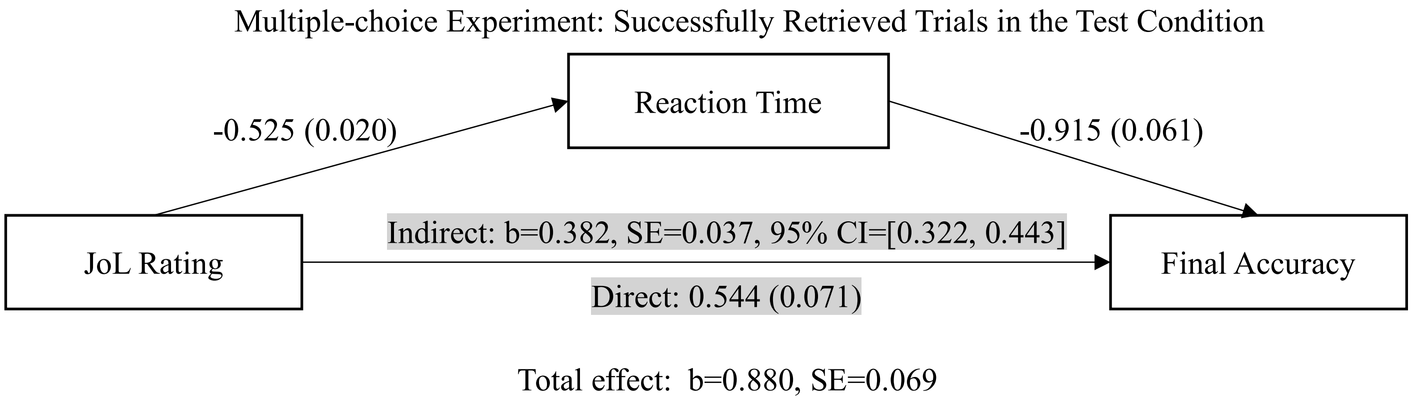

Supplement: Supplementary file 1 [file Table_1.pdf]
